# Identification of Novel Genomic-Variant Patterns of OR56A5, OR52L1, and CTSD in Retinitis Pigmentosa Patients by Whole-Exome Sequencing

**DOI:** 10.3390/ijms22115594

**Published:** 2021-05-25

**Authors:** Ting-Yi Lin, Yun-Chia Chang, Yu-Jer Hsiao, Yueh Chien, Ying-Chun Jheng, Jing-Rong Wu, Lo-Jei Ching, De-Kuang Hwang, Chih-Chien Hsu, Tai-Chi Lin, Yu-Bai Chou, Yi-Ming Huang, Shih-Jen Chen, Yi-Ping Yang, Ping-Hsing Tsai

**Affiliations:** 1 College of Medicine, Kaohsiung Medical University, Kaohsiung 807378, Taiwan; lintingyi2014@gmail.com; 2 Department of Ophthalmology, Taipei Veterans General Hospital, Taipei 112304, Taiwan; johnny19890821@gmail.com (Y.-C.C.); m95gbk@gmail.com (D.-K.H.); chihchienym@gmail.com (C.-C.H.); taichilin0331@gmail.com (T.-C.L.); suddenonset@gmail.com (Y.-B.C.); vghtpepmrcr@gmail.com (Y.-M.H.); sjchen@vghtpe.gov.tw (S.-J.C.); 3 College of Medicine, National Yang-Ming Chiao-Tung University, Taipei 11217, Taiwan; yj1007hsiao@yahoo.com; 4 Department of Medical Research, Taipei Veterans General Hospital, Taipei 11217, Taiwan; g39005005@gmail.com (Y.C.); cycom1220@gmail.com (Y.-C.J.); hernywe0228@gmail.com (J.-R.W.); chingderek@yahoo.com.tw (L.-J.C.); 5 Institute of Pharmacology, National Yang-Ming Chiao Tung University, Taipei 112304, Taiwan; 6 School of Medicine, National Yang-Ming Chiao Tung University, Taipei 112304, Taiwan; 7 Big Data Center, Taipei Veterans General Hospital, Taipei 112201, Taiwan; 8 Institute of Clinical Medicine, National Yang-Ming Chiao Tung University, Taipei 112304, Taiwan; 9 Department of Internal Medicine, Taipei Veterans General Hospital, Taipei 112201, Taiwan; 10 Critical Center, Taipei Veterans General Hospital, Taipei 112201, Taiwan

**Keywords:** inherited retinal dystrophies, whole-exome sequencing, retinitis pigmentosa

## Abstract

Inherited retinal dystrophies (IRDs) are rare but highly heterogeneous genetic disorders that affect individuals and families worldwide. However, given its wide variability, its analysis of the driver genes for over 50% of the cases remains unexplored. The present study aims to identify novel driver genes, disease-causing variants, and retinitis pigmentosa (RP)-associated pathways. Using family-based whole-exome sequencing (WES) to identify putative RP-causing rare variants, we identified a total of five potentially pathogenic variants located in genes OR56A5, OR52L1, CTSD, PRF1, KBTBD13, and ATP2B4. Of the variants present in all affected individuals, genes OR56A5, OR52L1, CTSD, KBTBD13, and ATP2B4 present as missense mutations, while PRF1 and CTSD present as frameshift variants. Sanger sequencing confirmed the presence of the novel pathogenic variant PRF1 (c.124_128del) that has not been reported previously. More causal-effect or evidence-based studies will be required to elucidate the precise roles of these SNPs in the RP pathogenesis. Taken together, our findings may allow us to explore the risk variants based on the sequencing data and upgrade the existing variant annotation database in Taiwan. It may help detect specific eye diseases such as retinitis pigmentosa in East Asia.

## 1. Introduction

Retinitis pigmentosa (RP, OMIM_268000) initially manifests with night blindness that develops to progressive visual-field loss [1]. Although unique pathways have been associated with different RP-phenotypes, they converge with irreversible photoreceptor dysfunction and apoptosis reflected in outer-nuclear-layer-thickness thinning and fundus pigment deposition [2]. RP manifests with rod photoreceptor dysfunction before the cone photoreceptor degeneration. Rod death reduces oxygen consumption by the cells, resulting in excessive oxygen levels in the outer retina, which overwhelms the antioxidant defense system and contributes to the cone cells’ loss of function and death. Rod cell death causes night blindness, whereas visual disability and blindness result from the later stages of cone degeneration. The inheritance pattern of RP includes 15–20% autosomal dominant (adRP), 20–25% autosomal recessive (arRP), 10–15% X-linked (XLRP), and the remaining 40–55% present with no family history. RP affects about 1 in 4000 persons worldwide [3,4,5], accounting for almost half of the inherited retinal dystrophy (IRD) spectrum [6]. Despite the advances in diagnostic techniques over the last decades, knowledge on the genetic architecture of IRD remains incomplete. RP is a complex disease for geneticists and physicians to decode as it presents significant genetic, clinical, and penetrance variability that shares overlapping driver genes with other IRDs. The driver genes may be polygenic and confounded with modifier genes, co-inheritance, age, and sex [7], yielding disperse findings that pose indefinite challenges in genetic studies/diagnosis/counseling.

Although some diseases are caused by mutations in a relatively small number of genes, the more prevalent IRDs are highly heterogeneous with many causative genes. RP is an extreme example, with associated mutations identified in at least 80 different genes [8,9,10,11]. These known RP-associated genes encode proteins responsible for a wide range of functions, such as rod-specific phototransduction (RHO; OMIM_180380), transcriptional regulation of photoreceptor differentiation (NRL; OMIM_162080), pre-mRNA splicing (PRPF31; OMIM_606419), and ciliary function (C2orf71; OMIM_613428). Among them, the EYS (OMIM_612424) [12], RP1L1 (OMIM_608581) [13], and CYP4V2 (OMIM _608614) [14] are found to be the most causative variants among the Asian (specifically, Japanese) patients [15], whereas RHO [16,17,18] and USH2A [19] are the most prevalent genes identified in Caucasians RP studies. However, these genes account for only approximately 60% of RP cases [1]. Further identification of novel genes is indispensable to provide valuable information for the diagnosis, prevention, and treatment of RP.

Extensive unknown RP genes, coupled with the lack of representation of Taiwanese gene frequencies, signify that an unbiased next-generation sequencing (NGS) using the whole-exome sequencing (WES) technique is necessary to overcome the limitations of molecular diagnosis via traditional screenings. For example, the current screening methods, such as panels and microarrays, assume that every SNP tested is independent, resulting in spurious associations to survive multiple testing corrections. Additionally, GWAS is subjected to missing heritability when a portion of genetic variance cannot be explained by all significant SNPs, often resulting from an incomplete linkage between causative variants and those genotyped. This becomes problematic when identifying variants of small effect or in small heterogeneous samples [20]. Whole-exome sequencing (WES), on the other hand, is an efficient method developed to overcome these limitations, particularly for monogenic inherited disorders, such as IRDs. So far, IRDs have been associated with more than 260 genes [8]; however, it is estimated that these genes account for only about 80% of the genetic diseases [21,22,23]. In a new era of IRDs’ gene identification, the introduction of next-generation sequencing that can combine molecular diagnostics, retinal disease gene identification, and an in silico functional assessment is important to help identify these remaining 20–30% of the novel and rare variants.

Thus, this study used WES with intrafamilial comparisons between the potential deleterious variants and neutral, unaffected families to identify RP disease-causing variants (DCVs) and correlate their genotypes and phenotypes. The majority of the cases were first screened by Sanger sequencing for variants in most likely candidate genes and then subjected to whole-exome sequencing. Additional family members were also recruited to perform segregation analysis of the mutation with the disease phenotype. Here, we summarize the recent repertoire of de novo variants and genes detected from WES family-based studies in the Taiwanese non-syndromic RP family and provide a detailed overview of the co-inherited/segregated cluster of novel genes and variants. By presenting examples of these cases, we highlight the challenges and limitations of WES data analysis and their implications for future genetic diagnostic procedures.

## 2. Results

### 2.1. Clinical Diagnosis Identifies Five RP Patients Out of a Family of Fourteen

**Figure 1:**
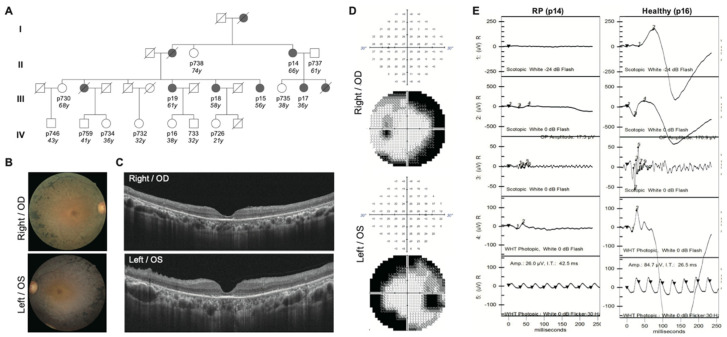
Representative results following ophthalmological features of the probands. (**A**) Pedigrees of RP. A total of 14 individual cases were recruited and are numbered in the pedigrees. Five were diagnosed as RP (p14, p15, p17, p18, and p19), shown as the filled box. The filled square (male) or circle (female) represents indicated RP patient, unfilled square (male) or circle (female) represents healthy individuals, and square (male) or circle (female) with slash represents deceased individuals. (**B**) Fundus photographs of the right (OD) and left (OS) eyes of the patient centered on the inferotemporal vascular arcade. At age 66, fundus photographs of this individual showed typical RP-associated characteristics, including attenuation of the retina blood vessels, bone spicule-like deposits, and waxy pallor of the optic disc. (**C**) OCT scans of the OD and OS eyes. (**D**) VF of both eyes. (**E**) ERG results of the normal and RP eyes. Compared to the normal case, electroretinograms of RP showed no detectable rod and cone responses.

The proband (p14) was a 66-year-old female RP patient whose symptoms occurred in her adulthood. A total of 14 individuals, who are either her close or distant relatives, were recruited for further genetics sequencing. Among them, five individuals were diagnosed with RP. The ophthalmic assessment, including the fundus photography, Optical Coherence Tomography (OCT), Visual field (VF), and Electroretinography (ERG) test, was performed at the Taipei Veterans General Hospital. The patient’s fundus photographs showed typical RP phenotypes, including the bone-spicule pigmentation in the peripheral retina, waxy pallor optic disc, and attenuation of retinal vessels (Figure 1B). The OCT analysis presented the bilateral diffuse disappearance of the photoreceptor layer outside of the fovea and mild epiretinal membrane formation (Figure 1C). The visual-field test indicated a right scotoma in the visual field’s mid-periphery (Figure 1D). The ERG analysis showed all ERG responses are markedly subnormal, with rod ERGs more affected than cone ERGs (Figure 1E).

Detail clinical features were recorded and are shown in Table 1. Among the 14 individuals, female females were RP patients. The average age of onset was 42.8, ranging from 29 to 55 years old. All five patients showed typical RP fundus features, with three showing severe bilateral macula involvement of disease and two of them showing only mild bilateral involvement. Though other ocular conditions might impact the visual acuity, the affected patients, especially those with macula involvement, showed worse performance. A global index based on the aggregated percentage of visual functions was seriously affected in RP patients. The family denied consanguineous marriage, and all affected members presented with classic RP findings are diagnosed with comprehensive ocular examinations (Table 1). In the cohort enrolled in this study, only females are affected (Figure 1A).

### 2.2. Whole-Exome Sequencing Identifies Potential Disease-Causing Variants

**Figure 2:**
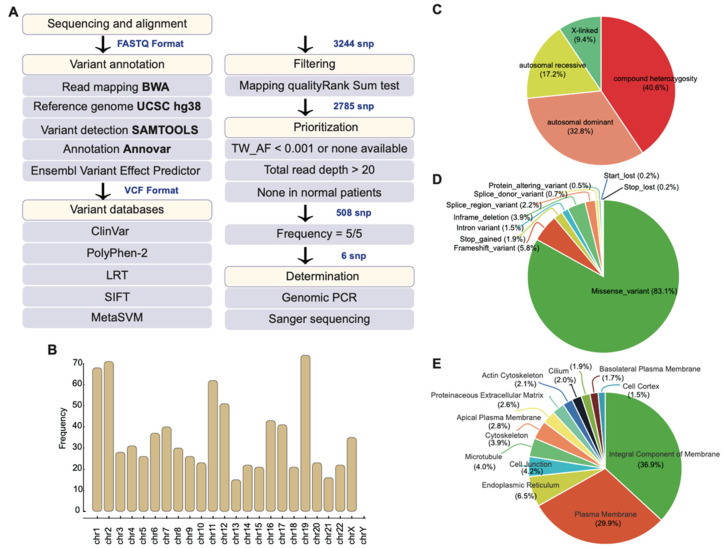
The general characterization of variants in the RP family. (**A**) Summary of analysis pipeline used in this study, including the variant annotation, filtering, and determination for pathogenicity variants. (**B**) The chromosome distribution of all variants in the RP family. (**C**) The proportion of inheritance modes of the variants. (**D**) The molecular consequence of all variants. (**E**) The cellular location of affected protein by variants.

The DNA of affected and non-affected individuals was extracted from blood samples. Exomes were captured and amplified based on SureSelect Human All Exon V6—Agilent. WES was performed on the Illumina HiSeq platform achieved read lengths of 100 bp. Reads/signals were defined as positive if they achieved read 20 quantity reads. After applying the filtering and data quality check workflow (Figure 2A), as described later in the Materials and Methods, we obtained raw data for identifying the genetic variants. The output raw reads (FASTA) were mapped to the reference genome (hg38), using BWA (BAM). Then, 3244 SNPs underwent variant calling, performed with GATK SAMtools, and annotated with the Ensembl Variant Effect Predictor (VEP), obtaining 2785 SNPs. The Wilcoxon rank-sum test was then applied to indicate that alternate alleles achieved higher mapping quality than reference alleles, reducing the SNPs’ numbers from 2785 to 508. The variants were further annotated by using Mutation Annotation Format (MAT), where the exonic functions and deleterious functions were further filtered and prioritized with the criteria: TWBK_AF < 0.001 or non-available and frequency = 5/5. This indicates that all affected rare variants were only presented in affected individuals and that these are mutations for identifying high penetrant variants. A total read depth of >20 was maintainedto ensure high quality in the SNPs found. Finally, a total of six rare variants were selected as functional candidate DCVs (Table 2). 

### 2.3. Mutational Profile of the Family

The distribution of 508 SNPs spanned across all chromosomes, except the Y chromosome, as only females were affected in the family. Chromosomes possessing the highest frequency of SNP were 1, 2, 11, 12, and 19, and chromosome X also presented with significant frequency and showed the presence of X-linked variants (Figure 2B). The total SNPs accounted for the compound heterozygous (40.6%), autosomal dominant (32.8%), autosomal recessive (17.2%), and X-linked (9.4%) inheritance modes (Figure 2C). The non-synonymous nucleotide polymorphism was predicted to cause deleterious consequences with missense (83.1%), frameshift (5.8%), stop-gain (1.9%), intron variant (1.5%), splice region variant (2.2%), splice donor variant (0.7%), protein altering variant (0.5%), start lost (0.2%), and stop lost (0.2%) (Figure 2D). These consequences had an impact on genes that are responsible for an integral component of membrane (36.9%), plasma membrane (29.9%), endoplasmic reticulum (6.5%), cell junction (4.2%), cytoskeleton (3.9%), and apical plasma membrane (2.8%) (Figure 2E). The enriched location at the membrane may result in impaired signaling transduction in the RP development. 

### 2.4. Gene Ontology of 508 Candidate SNPs and Their Associated Biological Function

**Figure 3:**
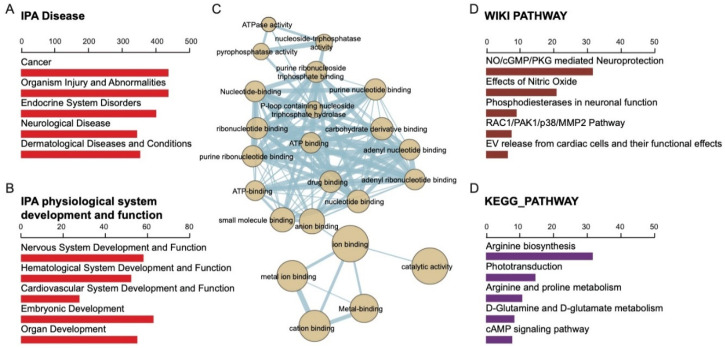
The biological characterization of RP-associated variants. The function of affected genes was mapped according to the QIAGEN Ingenuity Pathway Analysis, in the categories of the (**A**) Disease and (**B**) Physiological system development and function. (**C**) The regulation network of all altered genes in the RP family. The circle size presents the number of involved genes. The string shows the direct regulation. (**D**) Wiki pathway and KEGG pathway. This analysis shows the possible pathway of altered genes.

Systematic analysis of all SNPs (synonymous and non-synonymous) was enriched with gene ontology (GO). The genes were primarily associated with metabolic, disease-causing, signal-transducing, and immune-system-related functions. (Figure 3A,B). In particular, coverage of altered genes in human signaling and within the signal transduction domain signaling, G-protein-coupled receptors (GPCR) and receptor tyrosine kinase were enriched. Among these pathways, the enriched G alpha signaling event included visual phototransduction, inactivation, recovery, and regulation, and phototransduction cascade regulating receptor tyrosine kinase (Figure 3A). These events corresponded to phenotypic abnormalities, such as the thin retinal outer layer, abnormal rod electrophysiology, abnormal eye physiology, decreased retinal photoreceptor, and abnormal cone electrophysiology based on the MGI Mammalian Phenotype Ontology [24], as well as that of retinitis pigmentosa, bony spicule pigmentary, photophobia, abnormality of macular pigmentation, and cone-rod dystrophy based on the Human Phenotype Ontology [25] (Figure 3B). The altered genes in the RP family directly regulated each other in a regulation network. The more significant genes are involved in ion binding, catalytic activity, metal ion binding, cation binding, and metal binding (Figure 3C). Based on the WIKI pathway, the systemic functions of the altered genes included NO/cGMP/PKG mediated neuroprotection, effects of nitric oxide, phosphodiesterase in neuronal function, RAC1/PAK1/p38/MMP2 pathway, and EV release from cardiac cells [26]. Based on the KEGG pathway, they included arginine biosynthesis, phototransduction, arginine and proline metabolism, D-glutamine and D-glutamate metabolism, and the cAMP signaling pathway [27] (Figure 3D).

### 2.5. Highly Penetrant Disease-Causing Variants with High Frequency

**Figure 4:**
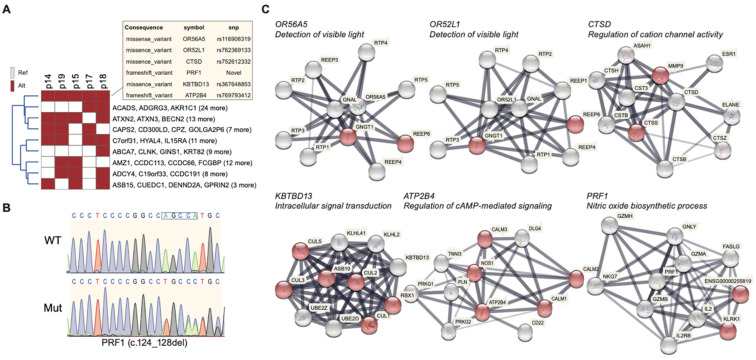
The molecular characterization of RP-associated variants. (**A**) Heatmap of carried variants in the RP family. The cell in red indicates the patient having variants, whereas the cell in white indicates the patient without variants. We labeled the gene locus of variants in the right of the row. (**B**) Identification of a mutation in the PRF1 gene. Sanger sequencing of p14 (Mut) and p16 (WT) confirmed a novel variants of c.124_128del (p.Trp42GlyfsTer34). (**C**) The PPI network of affected protein in the RP family. The network shows all interacted proteins with indicated protein. Among the interacted protein, the node in red represents the major proteins contribute to retina and signaling-associated biological function. The string represents the direct interaction with indicated protein.

To find the crucial and common variants from the RP family, we focused on targets with the highest frequency (5/5, affected/total cases). In the heatmap scheme, OR56A5, OR52L1, CTSD, PRF1, KBTBD13, and ATP2B4 were found at the top of the map (Figure 4A). In the 508 variants, only six variants were consistent among the RP patients, which indicates that the common variant may be crucial in the pathogenesis. OR56A5 (Olfactory Receptor Family 56 Subfamily A Member 5) is a protein-coding gene related to the signaling by GPCR and olfactory transduction pathways for G-protein-coupled receptor activity and olfactory receptor activity. Diseases associated with OR56A5 include Acrofacial Dysostosis 1, Nager Type, and Acrofacial Dysostosis. Olfactory receptors share a seven-transmembrane domain structure with many neurotransmitters and hormone receptors and are responsible for the recognition and G-protein-mediated transduction of odorant signals. Notably, the olfactory receptor gene family is the largest in the genome. The other gene we found, the OR52L1 (Olfactory Receptor Family 52 Subfamily L Member 1), belongs to the same gene family and is associated with diseases such as Pseudobulbar Palsy. Olfactory receptors interact with odorant molecules in the nose to initiate a neuronal response that triggers the perception of a smell [28]. 

Cathepsin D (CTSD) is a protein-coding gene responsible for making the protease enzyme CTSD, which modifies proteins by cutting them apart. CTSD is active in lysosomes to break down, activate, and regulate apoptosis of certain proteins. Diseases associated with CTSD include Ceroid Lipofuscinosis and Neuronal Ceroid Lipofuscinosis 10. Among its related pathways are peptide hormone metabolism and degradation of the extracellular matrix. An important paralog of this gene is ENSG00000250644, which encodes a member of the A1 family of peptidases. The encoded preproprotein is proteolytically processed to generate enzymes that exhibit pepsin-like activity and play a role in protein turnover and the proteolytic activation of hormones and growth factors. Mutations in this gene have been found to play a causal role in neuronal ceroid lipofuscinosis-10 and may be involved in the pathogenesis of other diseases, including breast cancer and Alzheimer’s disease [28].

KBTBD13 (Kelch Repeat and BTB Domain Containing 13) encodes a family of proteins that are involved in transcription regulation, ion channel tetramerization, and gating, protein ubiquitination or degradation, and cytoskeleton regulation. Diseases associated with KBTBD13 include Nemaline Myopathy 6 and Childhood-Onset Nemaline Myopathy. Its related pathways include Innate Immune System and Class I MHC mediated antigen processing and presentation. An important paralog of this gene is KLHL5. This family of genes contains a BTB domain involved in protein–protein interaction, such as self-association and the kelch repeats involved in transcription regulation, ion channel tetramerization, and gating, protein ubiquitination or degradation, and cytoskeleton regulation. The exact function of this family member has yet to be determined though [28].

ATP2B4 (ATPase Plasma Membrane Ca^2+^ Transporting 4) is a gene that encodes the calcium/calmodulin-regulated and magnesium-dependent enzyme that catalyzes the nucleotide binding and hydrolysis of ATP, coupled with the transport of calcium out of the cell [29]. It also plays a role in sperm motility by regulating sperm cell calcium homeostasis. Diseases associated with ATP2B4 include X-Linked Cerebellar Ataxia and Malaria. Its related pathways include Response to elevated platelet cytosolic Ca^2+^ and cGMP-PKG signaling pathways. An important paralog of this gene is ATP2B1. The protein encoded belongs to the family of P-type primary ion transport where ATPases are characterized by the formation of an aspartyl phosphate intermediate during the reaction cycle. 

The PRF1 gene, found in T cells and natural killer (NK) cells which destroy other cells, is one of the genes in which we found variants. It provides instructions for making perforin, a protein that is involved in the cytosis and in regulating the immune system. This deletion mutation results in an overall frameshift, leading to an abnormal reading frame from the 32nd amino acid onwards. As seen from results from the Sanger sequencing (Figure 4B), the c.124_128del mutation resulted in a loss of base pairs AGCCA that can be found in the wild type. This leads to the conversion of the 42nd amino acid tryptophan into glycine instead, which is projected to result in severe implications in protein expression. 

Since these variants are first identified in RP, few studies are about the association between the affected gene and RP. For seeking the possible association between RP, we use a protein–protein interaction database to support the establishment of an association. We used the STRING database to identify the possible interacting protein of indicated gene. The node in red represented the major proteins that contributed to the labeled retinal biological function among the interacted protein. In the protein–protein interaction analysis, OR56A5 and OR52L1 are involved in detecting visible light, cooperating with GNGT1 and REEP6. CTSD may cooperate with MMP9 and CTSS in the regulation of cation channel activity. KBTBD13 may cooperate with CUL1, CUL2, CUL3, CUL5, ASB10, and RBX1 in intracellular signal transduction. ATP2B4 may cooperate with CALM1, CALM2, CALM3, and NOS1 in the regulation of cAMP-mediated signaling. PRF1 may cooperate with KLRK1 and ENSG00000255819 in the Nitric oxide biosynthetic process (Figure 4C). According to the variant annotation by SIFT, PolyPhen2, LRT, and MetaSVM, two of the variants were predicted to be benign with tolerable consequences (OR56A5 and OR52L1). CTSD, KBTBD13, and ATP2B4 were reported as deleterious or possibly damaging, which implies the three genes may cause considerable effect. Although the novel variant of PRF1 lacked relevant data, frameshift deletion on PRF1 was still be expected to impair the biological function greatly.

The only variant that was ClinVar-annotated as pathogenic, CTSD (rs752612332), is associated with the regulation of proteolytic activity of the lysosomes. In neurons, loss of CTSD leads to lysosomal dysfunction and accumulation of different cellular proteins implicated in neurodegenerative diseases. While three of these six variants (50%), namely CTSD, KBTBD13, and ATP2B4, had been previously associated with RP, three are novel variants (50%) that have not been associated with RP (OR56A5, OR52L1, and PRF1). It is noteworthy that we found a novel variant of the PRF1 gene among the five RP patients. Meanwhile, it is not reported by other known databases (Table 3). 

## 3. Discussion

Defining ophthalmic disorders with a molecular genetic basis profoundly affects the understanding of genetic architecture for precise disease classification and gene-therapeutic development. Nevertheless, Taiwan often falls short of the screening process despite its population’s genetics being relatively homogeneous. As a result, the pool of RP-associated genetic populations is internationally underrepresented [30,31,32,33,34,35,36]. Attempting to characterize RP driver genes by using GWAS had proven limited as common variants tend not to identify the causal driver gene’s adequate fraction [37,38]. Their associations could only explain a small portion of heritability [39,40,41]. Thus, researchers began to diverge on the spectrum of whether the DCVs are presented as rare variants of large effect instead of common variants of small effect. 

The identification of rare SNPs by using a large population-based approach is usually hindered by the diverse genetic background and the requirement of a large-size population. The identification of rare variants should not be obtained via the simplex extension of common variant derivation methods using population-based sequencing [42], as it may conceal/overshadow the allele frequency of rare variants at the diluted population pool. A family-based approach using extended pedigrees that increases the founder effect and enriches the allele frequency of rare variants in the cohort [37] can be an alternative strategy to identify the putative SNPs. In this study, we used the family-based analysis to identify rare retinitis pigmentosa–related SNPs; we discarded those SNPs identified in most family members with normal phenotypes and further identified those putative rare SNPs that are associated with retinitis pigmentosa phenotypes. Rare variants may allow the hypothesis-free exploration of functional hypotheses [43] and the impact of rare variants on gene function and expression [42], and they serve as better drug candidates with genetically supported targets [44,45,46]. 

We identified a family of four generations for our study, where RP traits were prominent, with nine affected individuals (five living) diagnosed after thorough clinical examinations (Figure 1). Of the sixteen living members, we recruited fourteen to perform sequencing, of which five were diagnosed with RP. As this family presented with frequent RP diagnosis, we hypothesized that the disease alleles ran at a high frequency with a substantial portion of heterozygote carriers. Autosomal recessive inheritance was noted in patients p19 and p18, in which all affected individuals gave birth to unaffected children. In p14, both affected individual’s parents and children were affected, suggesting a different inheritance pattern. Interestingly, all affected individuals were females and beyond 32 years old, implying that the penetrance of DCVs is both sex-dependent and age-dependent. Based on the above pedigree analysis, our cohort’s inheritance pattern remained to be confirmed as the fourth generation had yet to reach the onset age.

In our in-house developed WES pipeline, we defined unaffected individuals as neutral backgrounds and filtered variants of affected individuals against those of their unaffected parents. As we removed variants present in non-affected members, we identified only high penetrant variants. We excluded DCVs with incomplete penetrance or were within carriers of the mutation who did not present the disease phenotype (Figure 1 and Figure 2A). Enriching all 508 SNP (synonymous and non-synonymous) to numerous ontologies in terms of biological ontology (BO), both WIKI and KEGG enriched pathways analysis indicated that these variants associated with NO/Arginine metabolism, NO/cGMP/PKG pathways known for neurite growth/protection/development vital for the neurosensory retina’s homeostasis/maintenance (Figure 3C). Various gene mutations of IRD share the common denominator of cell death via the excessive accumulation of cGMP in photoreceptors [47,48]. The nitric oxide (NO)–cGMP signaling pathway plays a role in neuronal structural plasticity where axonal retraction, neurite extension, and formation of presynaptic varicosities can be activated in photoreceptors following retinal detachment/regeneration [48]. NO, and cGMP stimulate neuritic sprouting dependent on Ca2+ influx through cGMP gated channels and PKG in cones [48,49]. The opposite was observed in rods where the pathway inhibited neurotic growth in a dose-dependent manner [48]. Perhaps this explained how cones were affected in RP more than rods initially. 

After assessing the overall 508 SNP profile, we then explored the cluster of SNPs presented in all affected individuals to highlight the most relevant and contributive driver genes for the family’s inheritance. Familial disease-associated mutations with a high frequency of 5/5 (100%) were found in all five family members analyzed. These included mutations in the genes OR56A5, OR52L1, CTSD, PRF1, KBTBD13, and ATP2B4. These six variants were all non-synonymous variants where OR56A5, OR52L1, KBTBD13, ATP2B4 are missense, and among the missense variants KBTBD13, ATP2B4 were predicted in silico to be deleterious. As the in silico evaluation of the missense variants may not be entirely accurate, we carefully surveyed the in silico predicted benign variants. OR56A5 and OR52L1 are olfactory receptors that belong to a large family of GPCR that share a 7-transmembrane domain structure many neurotransmitters and hormone receptors that are responsible for recognizing G-protein-mediated transduction of odorant signals—the two genes had not been reported with disease. Examining our variants rs116908319 c443C>T (OR56A5) corresponded to a missense variant at a highly conserved location Ala148Asp reported as a naturally occurring variant despite an unfavorable change of conserved amino acid properties [50]. The second olfactory receptor identified, rs762369133 (OR52L1) c317A>G, turned His106Pro. The highly conserved His residue is altered to variant protein residue with a rigid side chain restricting the protein’s conformation, leading to protein malfunction or dysfunction. 

After ruling out benign variants, we next explored the in silico predicted pathogenic variants KBTBD13 and ATP2B4. KBTBD13 belongs to a family of genes encoding proteins consisting of the BTB domain and several kelch repeats. The BTB domain serves as a protein–protein interaction module, where it aids self-association and non-self-associations, and the kelch motif has been reported with diverse functions. The family of genes is responsible for transcription regulation, ion channel tetramerization and gating, protein ubiquitination or degradation, and cytoskeleton regulation. Mutations in the gene KBTBD13 have been associated with nemaline myopathy 6, characterized by childhood onset of progressive proximal muscle weakness. Our KBTBD13 variant identified, rs367648853, was a missense variant c958G>A, Val320Met, not a large alteration, retained aliphatic hydrophobic property. The change was not expected to result in a change in the protein’s function. On the other hand, ATP2B4 belongs to the family of P-type primary ion transport ATPases. The mammalian plasma membrane calcium ATPase removes bivalent calcium ions from eukaryotic cells against very large concentration gradients and plays a critical role in intracellular calcium homeostasis. Disease-associated with the gene encompasses X-linked cerebellar ataxia, malaria, Schnyder corneal dystrophy, long QT syndrome, and hereditary spastic paraplegia. A change from an Ile to a Val side chain was not a large one and may or may not change the protein’s function. The Ile-to-Val residue change only modifies the amino acid size without affecting the hydrophobicity, and this change is expected not to affect the protein’s function. 

Frameshift variants were assumed to be prone to loss-of-function consequences, and in our study, we identified two frameshift variants, CTSD and PRF1 (Table 3). CTSD encodes a pepsin-like protein that maintained protein turnover (intracellular protein breakdown) and proteolytic activation of hormones/growth factors. The encoded preprotein is cleaved to generate CTSD light and heavy chains that required heterodimerization to form the mature enzyme. Upon activation (binding to ADAM30 or GRN), CTSD activates lysosome and extracellular matrix degradation and APP degradation. The gene is located at chr11: 1,773,982-1,785,222 with the size of 412 aa, 44 kDa. These mutations in this gene are associated with Ceroid lipofuscinosis and neuronal ceroid lipofuscinosis, two lysosomal storage disorders that are involved in the pathogenesis of Alzheimer’s disease. The novel c.268dupC pathogenic variant located at chr11: 1759600-1759606 (GRCh38.p12) was associated in our RP patient generated a duplication that caused a frameshift at codon 90 through duplication of nucleotide (268) that resulted in Gln (90) changing to Pro via frameshift and generated a premature stop codon and terminates after 50 aa, denoted p. Gln90ProfsX50. The 412 amino acid protein was shorted to 140 amino acids. The DCV was predicted to cause loss of normal protein function through protein truncation or nonsense-mediated mRNA decay. As the only variant that was ClinVar-annotated as pathogenic, the CTSD variant was reported previously as a homozygous change in two siblings with myoclonic epilepsy, respiratory failure CTSD deficiency [51]. The gene (variant c.57_63del and c.1064C > T) had been previously reported in a 45-year-old male with adult-onset ataxia, and visual function had rapidly deteriorated from the age of 32. Ophthalmological examination showed an RP-like phenotype with central involvement. Based upon the ataxia/RP complex, exome sequencing prioritized two previously unreported compound heterozygous variants in CTSD. In patient-derived fibroblasts, biochemical analyses indicated that mutant CTSD is produced but not efficiently processed into the mature form, linked to autophagic dysfunction, may predispose neurodegenerative diseases in late adulthood. OR56A5, OR52L1, and CTSD were located on chromosome 11 location 5,968,052, 5,986,614, and 1,759,599, respectively. Nevertheless, more studies into linkage disequilibrium are needed to confirm whether the locus was inherited together (Table 2).

Our novel variant identified in perforin 1 (PRF1) encodes complement component C9 structural similar protein that assembles into membrane pores in response to virus infection and cancer. Upon calcium ion binding, the protein is inserted into the target cell’s membrane and oligomerizes to form pores that uptake cytotoxic granzymes and undergo secretory granule-dependent cell death. The gene is located at chr10q22.1 and encodes for 555 aa, 61 kDa. Mutations in this gene were associated with multiple sclerosis, lymphoma, autoimmune lymphoproliferative syndrome (ALPS), aplastic anemia, and familial hemophagocytic lymphohistiocytosis type 2 (FHL2). c.124_128del cause frameshift and terminated after 34 amino acids at the protein PRF1, significantly shortening the original 555 amino acid protein to 76 amino acids. This DCV was predicted to cause loss of normal protein function through protein truncation or nonsense-mediated mRNA decay. PRF1 was associated with the crucial biological function of the nitric oxide biosynthesis pathway (Figure 4C). This was consistent with the abovementioned pathways enriched from the entire familial nucleotide polymorphism library (508 variants) (Figure 3D). In other words, it pinpointed the possible disease-causing pathway, which accentuates the role of the dysregulated NO metabolism pathway in the familial RP phenotype. However, they lacked enough supporting evidence under the assumption that the presence of any rare variant increases the disease risk given no minor alleles in a region. These three rare variants were of utmost importance in RP-affected members of this family. It was prominent and was presented in high frequency in the affected members’ genetic pool versus non-affected. 

Attempting to establish genotype–phenotype correlations, we found that intra-familial heterogeneity was age-independent, suggesting additional involvement of modifiers and contributive genes outside the six variants we have identified. To assess this, we grouped affected members of similar age and compared their clinical phenotypes. Comparing p14 (age 66) and p19 (age 61), we observe that although they are similar in macular involvement, pigment deposit, optic disc waxy, and artery attenuation, their VA is different. P19 retained/preserved a better VA (OD, OS: 0.7, 0.9 versus 0.4, 0.1). P19′s (age 61) VA preservation is similar to p17 (age 28), despite macular involvement and arterial attenuation. Comparing p15 (age 56) and p18 (age 58), although p18 suffered from the worst VA compared to p15 (OD, OS: 0.5, 0.3 vs. 0.6, 0.6), p18 presents with milder macular involvement, milder optic disc waxing, and milder macular involvement but significantly worse VA. The macular area involved (broad but light.) vs. severity (little involvement but severe degeneration) non-RP involvement: lifestyle gaming/studying, other ophthalmologic diseases DM-retinopathy, AMD, cornea, cataract, etc. Lifestyle, environmental influences apart from genetics. Despite the family’s clinical heterogeneity, the same six allelic truncated variants were shared between affected individuals of the same family suggested a polygenic role in forming the disease’s susceptibility. RP’s pathogenesis plaguing the family may not be straightforwardly caused by a mutant variant of a single gene but that of a complex disease, where genetic components had jointly contributed or interacted between polymorphic genes and other modifiers. The allelic combination of genes had emerged as a clinically significant and heterogeneous form. 

Genome-wide association studies that focus on detecting risk variants for gene discovery and precision medicine can predict disease risk for an individual. Taiwan Biobank owns the largest publicly available genetic database of individuals with East Asian ancestry [52] and has the unique sequencing chips focusing on healthy and subhealth populations as well as several disease cohorts. We identified RP-associated variants that greatly improved the coverage of SNP genotyping data. The data may help the design of a custom SNP array for genetic studies in the Han Chinese population accounts for one-fifth world’s population. To be compatible with the system in Taiwan Biobank, we will also subject our samples to the TWBv2 array analysis, which may allow us to explore the risk variants based on the sequencing data from Taiwan Biobank. In the near future, our designed sequencing chips may help detect specific eye diseases, such as retinitis pigmentosa, in East Asia. Overall, our study has explored comprehensive genetic information of Taiwan RP patients and can serve as a reference for precision health management. 

## 4. Materials and Methods

### 4.1. Samples Collection, Clinical Examination, and Genomic DNA extraction

Fourteen RP patients diagnosed between 2017 and 2019, at the Taipei Veterans General Hospital, were enrolled in the study. The RP family has five RP patients and nine normal controls. The inclusion criteria were patients who have underwent comprehensive ophthalmic examination, including visual acuity, visual field test, BCVA, slit-lamp examination, fundus photography, full-field electroretinogram (ERG), and spectral-domain optical coherence tomography (SD-OCT). The study was approved by the institutional review board of Taipei Veterans General Hospital (Institutional Review Board, Taipei Veterans General Hospital, IRB NO.: 2021-04-009A, 12 April 2021) and was conducted in accordance with the tenets of the Declaration of Helsinki. Informed consent was obtained from each study subject before recruitment. Peripheral blood samples were collected in every study subject, and the DNA samples were collected. Genomic DNA was extracted from peripheral blood using the Qiagen FlexiGene DNA Kit (Qiagen, Hilden, Germany) under standard procedures. 

### 4.2. Whole-Exome Sequencing

Genomic DNA of the entire subjects was extracted from whole blood, using the Qiagen Blood DNA Kit (Qiagen, Hilden, Germany) according to the manufacturer’s directive. The genomic DNA of four affected patients and unaffected family members from the RP family were subjected to WES analysis. The whole-exome was captured by using Agilent SureSelect Human All Exon Kit V6 (Agilent Technologies, Santa Clara, CA, USA). The HiSeq 2500 (Illumina, San Diego, CA, USA) platform was used for paired-end sequencing with reading lengths of 150 bp and used a minimum of 20 million reads per sample.

### 4.3. Variant Calling and Variant Annotation

Raw reads were aligned to hg38 with BWA MEM 0.7.17 [11]. We used GATK v4.1.4.1 and followed the GATK best practices workflows for variant discovering. The output variant call format (.vcf) files were annotated, using Ensembl Variant Effect Predictor (VEP) v99 with dbNFSP v4.0a. We further evaluated the pathogenicity of variants based on the prediction scores and its consequence. Variants with high-impact consequences, such as splice acceptor/donor, frameshift, and nonsense variants, were assigned with high pathogenicity. Missense variants that meet one of the following criteria were included for the analysis: PolyPhen (http://genetics.bwh.harvard.edu/pph/), likelihood ratio test (LRT), SIFT (https://sift.bii.a-star.edu.sg/), MetaSVM.

### 4.4. IPA Analysis, WIKI Pathway, and Gene Ontology

The post-GWAS pathway analysis approach was applied to the post-GWAS assigned genes. We used IPA (Qiagen, Hilden, Germany) and pathway analysis to identify the relevant pathway/gene network enrichments of these post-GWAS assigned genes after quality check and selection. The p-values represent the probability for each result and are corrected for the multiple testing (Benjamini–Hochberg method) that arises from evaluating the submitted list of genes against every pathway. We analyzed the canonical pathways and gene networks from the post-GWAS assigned genes. Ingenuity Pathway Analysis (IPA) was used to measure the statistical significance of the relationship pattern of the proteins produced by the genes studied here and matched with the prior published data [53]. IPA is based on experimentally validated pathway enrichments that include an upstream regulatory analysis of the genes.

We used WIKI pathway analysis to determine whether a prior defined set of genes and our gene list of interest displayed statistically significant, concordant alterations in gene expression associated with a disease that manifests at the level of biological pathways or co-regulated gene sets [26]. Pathway analysis uses prior gene sets that have been grouped together by their involvement in the same biological pathway [26]. The GO analysis was used to investigate the function of genes (corresponding proteins) in homo sapiens, their ontology, and the involved pathways [54]. The enrichments of post-GWAS genes in the GO biological process and cellular component were investigated.

### 4.5. Sanger Sequencing

Mutations discovered by next-generation sequencing were sequenced by using Sanger sequencing. The identified novel variants were proved by PCR and Sanger sequencing analysis in the remaining affected (p14, 15, 17, 18, and 19) and unaffected family members. PCR was performed in BioRad PCR machines with specific primers (Table 4). The PCR products were purified (Qiagen, Hilden, Germany) and sequenced by Tri-I Biotechnology Ltd. (Taiwan). The variant pattern was analyzed in the RP families. 

### 4.6. Statistical Analysis 

Statistical analyses were performed by using IBM SPSS Statistics version 22 (IBM Corp., Armonk, NY, USA). Categorical variables were compared between patients of different genotypes, using Fisher’s exact test or binary logistic regression analysis. Mann–Whitney U test or one-way ANOVA was applied to compare continuous variables. Multiple linear regression was performed to assess the contributions of major risk factors to RP phenotypes. Values of *p* < 0.05 were considered statistically significant. 

## 5. Conclusions 

In summary, we reported a novel mutation in gene PRF1 that causes RP, suggesting the instability/turnover defect in the retina as a possible mechanism for the disease. Our study provided valuable insight into the etiology of IRD and contributed toward a more comprehensive understanding of this heterogeneous group of disorders by cataloging novel causative variants. Cataloging national DCV engages with therapeutic design discussion and the invitation to the rare international disease-tackling community. The present study implicated novel candidate DCVs for RP, especially in the Taiwanese population, and contributes to an improved understanding of the biology/molecular basis of RP. 

## Figures and Tables

**Table 1:** Clinical feature of all 14 patients.

Patient	Gender	Diagnosis	Age	Age of Onset	RP Fundus Feature	Visual Acuity OD OS		Macular Involvement OD OS		Visual Field OD OS	
p14	Female	RP	66	43	Yes	0.4	0.1	Yes	Yes	-	-
p15	Female	RP	56	40	Yes	0.6	0.6	Yes	Yes	-	-
p17	Female	RP	36	29	Yes	0.8	0.6	Mild	Mild	-	-
p18	Female	RP	58	55	Yes	0.5	0.3	Mild	Mild	+	+
P19	Female	RP	61	47	Yes	0.7	0.9	Yes	Yes	-	-
P16	Female	Healthy	38	ND	No	1.0	1.0	No	No	+	+
P726	Male	Healthy	21	ND	No	1.0	1.0	No	No	++	++
P730	Female	Healthy	68	ND	No	0.8	0.7	No	No	++	++
P732	Female	Healthy	32	ND	No	1.0	1.0	No	No	+++	+++
P733	Male	Healthy	323	ND	No	1.0	1.0	No	No	+++	+++
P734	Male	Healthy	36	ND	No	1.0	1.0	No	No	+++	+++
P735	Female	Healthy	38	ND	No	1.0	1.0	No	No	+++	+++
P737	Male	Healthy	61	ND	No	0.8	0.9	No	No	++	++
P738	Female	Healthy	74	ND	No	0.8	0.8	No	No	++	++

OD: right eye, OS: left eye, RP: Retinitis Pigmentosa, -: VFI between 20-40%, +: VFI between 40-60%, ++: VFI between 60-80%, +++: VFI between 80-100% field, RP: Retinitis Pigmentosa, ND: non detected.

**Table 2:** Database annotation of six candidate snps in Retinitis Pigmentosa family.

Symbol	SnpDB	Chrom	Pos	Ref	Alt	Hgvsc	Consequence	Impact	Inheri-tance	Clin_ Sig	Polyphen	Lrt	Sift	Metasvm
OR56A5	Rs116908319	Chr11	5968052	G	A	NM_001146033.1:c.443C > T	missense variant	Mid	--	--	B	--	T	--
OR52L1	Rs762369133	Chr11	5986614	T	C	NM 001005173.3:c.317A > G	missense variant	Mid	--	--	B	N	T	T
CTSD	Rs752612332	Chr11	1759599	T	TG	NM_001909.5:c.268dup	Frameshift variant	High	AR	P	--	--	--	--
PRF1	Novel	Chr10	70600774	CAGCCA	C	NM 005041.5:c.124_128del	Frameshift variant	High	--	--	--	--	--	--
KBTBD13	Rs367648853	Chr15	65077773	G	A	NM 001101362.2:c.958G*A	Missense_variant	Mid	AD	--	B	--	D	T
ATP2B4	Rs769793412	Chr1	203707074	A	G	NM_001001396.2:c.1165A > G	Missense_ variant	Mid	--	--	PD	N	T	T

M: Moderate, AD: autosomal dominant, AR: autosomal recessive, Mu: multifactorial, P: Pathogenic; B: Benign, PD: Possibly damaging, Irt: Likelihood ratio test, sift: Sorting Intolerant.

**Table 3:** Related reference of candidate genes.

Consequence	Gene	Snp	p14	p15	p17	p18	p19	Retinitis Pigmentosa
Missense variant	OR56A5	Rs116908319	o	o	o	o	o	none
Missense variant	OR52L1	Rs762369133	o	o	o	o	o	None
Frameshift variant	CTSD	Rs752612332	o	o	o	o	o	10.1002/mds.28106
Frameshift variant	PRF1	Novel	o	o	o	o	o	None
Missense variant	KBTBD13	Rs367648853	o	o	o	o	o	10.1016/j.ajhg.2020.10.020
Missense variant	ATP2B4	Rs769793412	o	o	o	o	o	10.1080/13816810.2019.1703014

**Table 4:** Primers for sanger sequencing.

Gene	Forward (5′ to 3′)	Reverse (5′ to 3′)
ATP2B4	CCACTGTCTGTTCCCTATGC	CAGGGACTTCTGCTCTTGTG
CTSD	CCCTGCTGAGAGCAAGGACC	GACAGAAGCCAGGGGTCTAGA
KBTBD13	CTTCTGCTACGACCCCGAC	CCTCGATGGCGTAGAGCAG
OR52L1	GCCTATGATGGTGGCTTGG	GGAGGCTATCCCAGCCTTC
OR56A5	GGCCATATCTCCCTCACC C	GGAAGCCTCTCTGCACCAG
PRF1	CTTCAGTGGAGCTGACTTTG	GGGAAGGGAGCAGTCATC
